# Inhibition of lysosomal protease cathepsin D reduces renal fibrosis in murine chronic kidney disease

**DOI:** 10.1038/srep20101

**Published:** 2016-02-02

**Authors:** Christopher Fox, Pasquale Cocchiaro, Fiona Oakley, Rachel Howarth, Krystena Callaghan, Jack Leslie, Saimir Luli, Katrina M. Wood, Federica Genovese, Neil S. Sheerin, Anna Moles

**Affiliations:** 1 Fibrosis Research Group, Institute of Cellular Medicine, Newcastle University, Newcastle Upon Tyne, UK; 2 Department of Molecular Medicine and Medical Biotechnology, University of Naples Federico II, Italy; 3 Department of Cellular Pathology, Royal Victoria Infirmary, Newcastle Upon Tyne, UK; 4 Nordic Bioscience, 2730 Herlev, Denmark

## Abstract

During chronic kidney disease (CKD) there is a dysregulation of extracellular matrix (ECM) homeostasis leading to renal fibrosis. Lysosomal proteases such as cathepsins (Cts) regulate this process in other organs, however, their role in CKD is still unknown. Here we describe a novel role for cathepsins in CKD. CtsD and B were located in distal and proximal tubular cells respectively in human disease. Administration of CtsD (Pepstatin A) but not B inhibitor (Ca074-Me), in two mouse CKD models, UUO and chronic ischemia reperfusion injury, led to a reduction in fibrosis. No changes in collagen transcription or myofibroblasts numbers were observed. Pepstatin A administration resulted in increased extracellular urokinase and collagen degradation. *In vitro* and *in vivo* administration of chloroquine, an endo/lysosomal inhibitor, mimicked Pepstatin A effect on renal fibrosis. Therefore, we propose a mechanism by which CtsD inhibition leads to increased collagenolytic activity due to an impairment in lysosomal recycling. This results in increased extracellular activity of enzymes such as urokinase, triggering a proteolytic cascade, which culminates in more ECM degradation. Taken together these results suggest that inhibition of lysosomal proteases, such as CtsD, could be a new therapeutic approach to reduce renal fibrosis and slow progression of CKD.

The worldwide prevalence of chronic kidney disease (CKD) is estimated to be between 8–16% and is predicted to rise due to the ageing population and an increase in the incidence of diabetes and hypertension^1^. There are many causes of CKD including ischemic, toxic and infectious insults to the kidney and genetic, endocrine and immunological diseases. Progression of CKD results in end-stage renal disease (ESRD) and organ failure. Treatments to stop or slow the progression of CKD to ESRD are currently very limited^2^, with increasing numbers of patients requiring life-long dialysis or transplantation. Glomerulosclerosis and tubulointerstitial fibrosis are two main histological features of CKD. After kidney injury there is a physiological wound healing response to restore normal function and tissue homeostasis. However, repetitive insults or dysregulation of this response leads to excessive, pathological deposition of extracellular matrix (ECM) proteins such as fibrillar collagens (mainly type I and III), fibronectin and laminins. ECM deposition, crosslinking, turnover and degradation are finely regulated *in vivo* by proteases, transglutaminases, lysil oxidases and their inhibitors.

The study of protease biology is challenging at many levels: their regulation is complex occurring during gene transcription, cell trafficking, extracellular secretion, activation of latent forms and recycling; their substrate specificity and preference can vary from *in vitro* to *in vivo* and diseased to non-diseased tissues and finally there is a high degree of redundancy amongst different proteases, which can lead to complex compensatory mechanisms. There are two main families of proteases which have been implicated in the progression of renal fibrosis, metalloproteinases (MMP)^3^ and serine proteases^4^. However, the role of other proteases such as lysosomal cathepsins (Cts) is poorly understood in the context of renal fibrosis, despite playing an important role in other fibrotic diseases such as liver (CtsB), lung (CtsK) and heart (CtsL) fibrosis. CtsB inactivation attenuates hepatic damage^5^ and reduces scarring^6^,^7^ in several experimental models of liver fibrosis. In contrast in bleomycin lung fibrosis model, CtsK deficient mice have a worse outcome than wild type mice^8^, while transgenic overexpressing CtsK mice show a reduction in lung fibrosis^9^. Similarly, CtsL knock-out mice develop spontaneous age-related cardiac fibrosis^10^ while overexpression of human CtsL in a murine model of cardiac hypertrophy leads to an improvement of cardiac function and fibrosis^11^. Despite the evidence in other organs the role of lysosomal cathepsins in kidney fibrosis remains unclear. Therefore the aim of this study was to analyse the role of cathepsins in renal fibrosis. Here we describe a novel role for CtsD in kidney fibrosis. Screening of human kidney biopsies showed stronger CtsD staining in kidneys with tubular damage, localizing CtsD mainly in cytosolic vesicles of distal tubules. Analysis of aspartyl and cysteine cathepsins expression in mouse obstructive nephropathy showed an increase in CtsD and B but not L. Pharmacological inhibition of CtsD but not CtsB led to a reduction of kidney fibrosis in two different models of CKD, unilateral ureteric obstruction (UUO) and chronic ischemia reperfusion injury (IRI). Our *in vivo* and *in vitro* observations support a novel mechanism of action by which inhibition of CtsD leads to an impairment of lysosomal recycling increasing the amount of active proteases available in the extracellular space, such as UPA. Active UPA could then regulate and activate plasmin, enhancing ECM remodelling, ultimately reducing renal fibrosis.

## Results

### CtsD and B are differentially expressed in distal and proximal tubules respectively during human kidney disease

**Figure 1:**
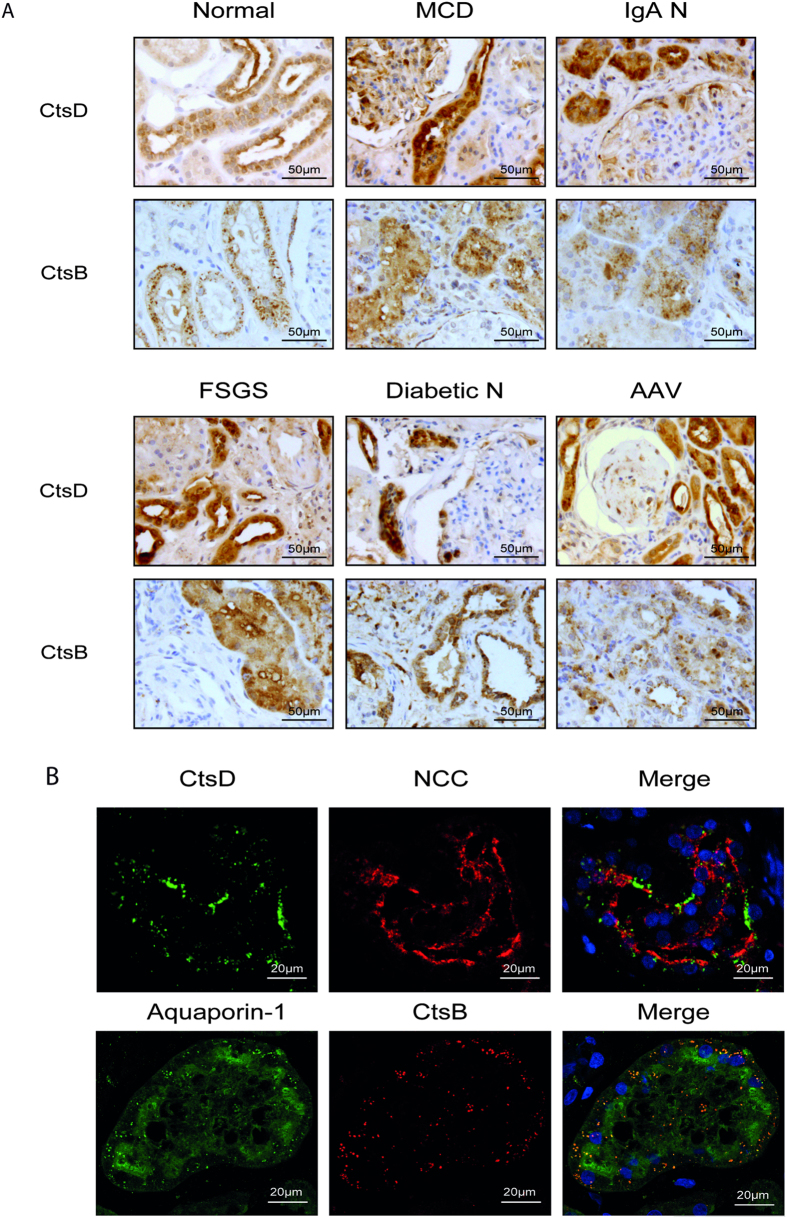
CtsD and CtsB expression in human normal and diseased kidneys. Representative pictures of CtsD or B staining in normal kidney, minimal change disease (MCD), IgA nephrophaty (IgA N), focal segmental glomerulosclerosis (FSGS), diabetic nephrophaty (Diabetic N), anti-neutrophil cytoplasmic antibody (ANCA) associated vasculitis (AAV) human biopsies **(A)**. A minimum of three different patient biopsies were stained per disease. Representative confocal microscopy pictures of NCC/CtsD or Aquaporin-1/CtsB dual immunofluorescence of human kidney biopsies **(B)**.

We determined the expression of CtsD or CtsB in normal human kidney and a range of human kidney diseases: minimal change disease (MCD), IgA nephropathy (IgA N), focal segmental glomerulosclerosis (FSGS), diabetic nephropathy (Diabetic N) and anti-neutrophil cytoplasmic antibody (ANCA) associated vasculitis (AAV). Analysis by a renal histopathologist identified a common expression pattern for all the diseases analysed, with CtsD or CtsB mainly expressed in cytosolic vesicles from distal or proximal tubular cells respectively (Fig. 1A). Interestingly, areas with a greater number of damaged tubules had more CtsD expression than unaffected areas or normal kidneys. No differences in CtsB expression were detected between normal and diseased kidneys (Fig. 1A). Of note, both CtsD and B were also detected in some podocytes and glomerular crescents. The distal or proximal tubular distribution of CtsD or B was also confirmed by confocal microscopy using thiazide-sensitive NaCl co-transporter (NCC) and aquaporin-1 as a distal or proximal tubular markers respectively (Fig. 1B). Based on these observations, CtsD could potentially play a role in tubular injury.

### Renal CtsD and B expression is induced in obstructive nephropathy

**Figure 2:**
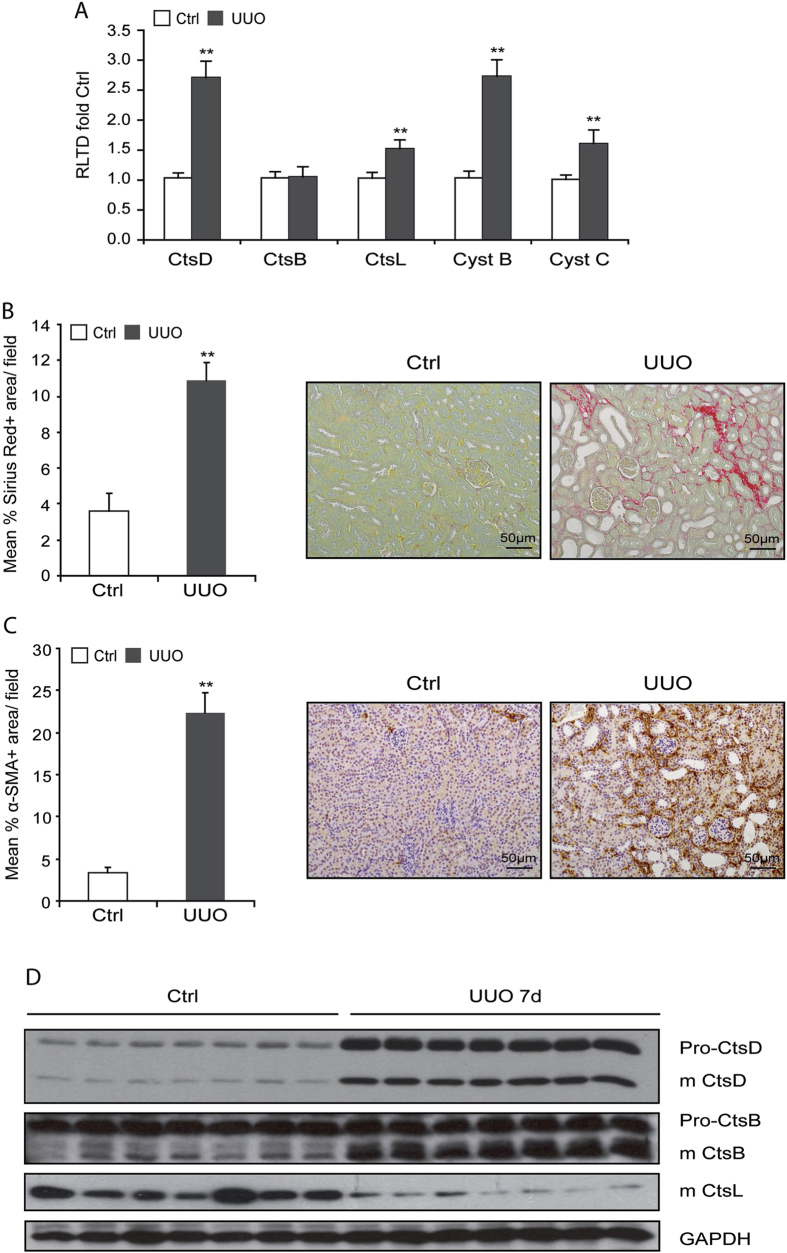
Aspartyl and cysteine cathepsin expression in 7 day UUO kidneys. CtsD, CtsB, CtsL, Cystatin B and Cystatin C mRNA expression **(A),** morphometric analysis of SR +ve **(B)** and α-SMA +ve **(C)** area/field and representative pictures of cortex from contralateral and 7 days UUO kidneys. Western Blot of pro and mature forms of CtsD, CtsB, CtsL and GAPDH **(D)** of contralateral and 7 days UUO kidney lysates. N = 7, t-test analysis, *P ≤ 0.05 or **P ≤ 0.01.

We next analysed aspartyl CtsD and cysteine CtsB and L expression in mice kidneys after 7 days Unilateral Ureteric Obstruction (UUO). CtsD, L and cysteine cathepsin inhibitors, cystatin B and C, mRNA expression were significantly increased in UUO kidneys compared to contralateral unobstructed kidneys, however, CtsB mRNA expression did not change (Fig. 2A). Cortical fibrosis in UUO kidneys was confirmed by morphometric analysis of Sirius Red and α-SMA (Fig. 2B,C).

Although cathepsins are regulated at the transcriptional level, the main step in regulating their activity occurs in the lysosomes where cathepsins are stored as inactive zymogens before being cleaved into the active protease^12^. Pro- and mature CtsD as well as mature CtsB were increased in UUO kidneys. In contrast mature CtsL decreased in diseased kidneys (Fig. 2D). To define the time course of CtsD upregulation, protein and mRNA levels were measured 5, 7 and 10 days after UUO induction. Both protein and gene expression were significantly upregulated by day 5 UUO with further increase by day 7. CtsD mRNA expression rose further by day 10 while protein expression plateaued after day 7 (Supplementary Fig. S1A,B). Therefore, during UUO there is a differential regulation of aspartyl and cysteine cathepsins with an increase of CtsD and B protein expression and processing into mature forms and a decrease in mature CtsL.

### Pepstatin A reduces kidney fibrosis in two different models of chronic kidney disease

**Figure 3:**
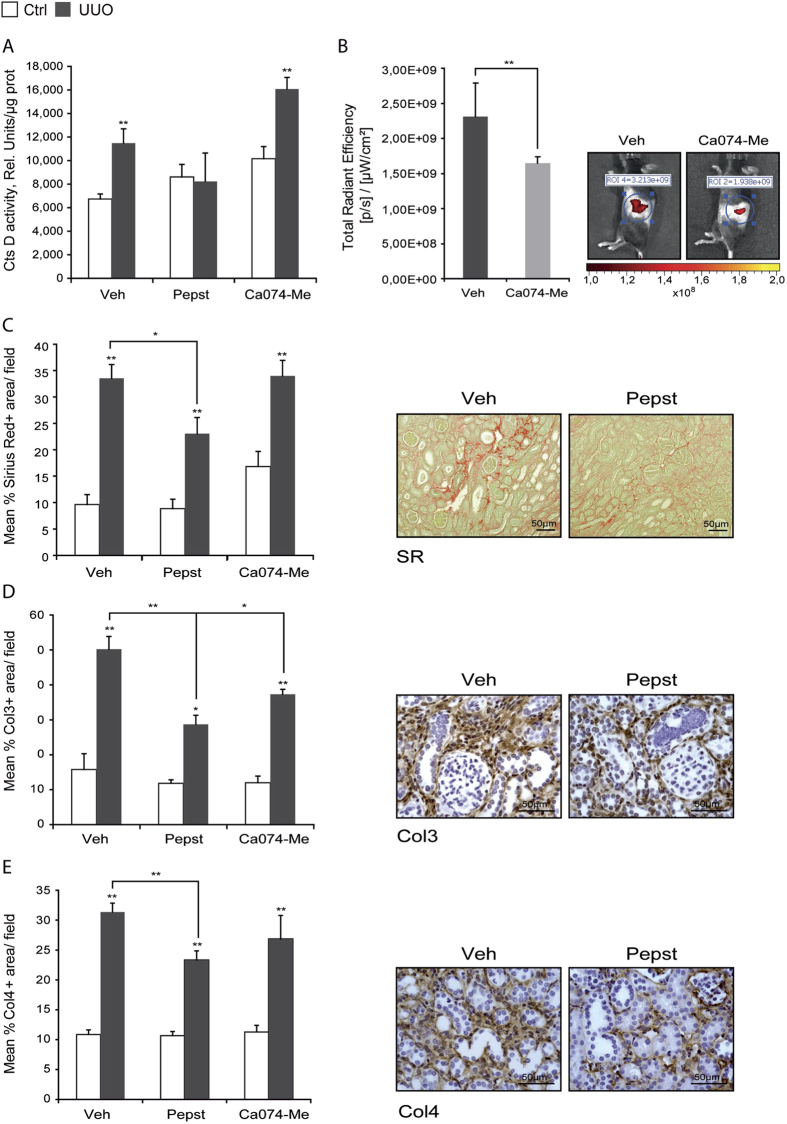
Cathepsin D inhibition but not B reduces cortical collagen deposition in 15 days UUO model. CtsD fluorometric activity in kidney lysates assessed by the cleavage of a specific fluorescently labelled substrate **(A)** IVIS assessed *in vivo* CtsB kidney activity in 10 days UUO **(B)**. Morphometric analysis and representative pictures of Sirius Red (SR) +ve **(C)**, Col3 +ve **(D)** and Col4 +ve **(E)** area/field in cortex of contralateral and day 15 UUO kidneys. Animals were treated with vehicle, Pepstatin A 20 mg/Kg or Ca074-Me 10 mg/Kg from day 5. N = 6, 1 way ANOVA,*P ≤ 0.05 or **P ≤ 0.01.

Active CtsD and B act as pro-fibrogenic enzymes in liver fibrosis^6^. To investigate whether the increase in active enzymes was playing a role in the development of kidney fibrosis, Pepstatin A (CtsD inhibitor) or Ca074-Me (CtsB inhibitor) were administered three times a week for 15 days following UUO. To be more representative of human disease, surgery was performed and renal injury was allowed to develop for 5 days before starting treatment, by which stage an increase in α-SMA and Col1A1 gene expression was already evident in untreated UUO mice (Supplementary Fig. S1C,D). Consistent with Fig. 2D, CtsD activity was significantly increased in injured kidneys and reduced back to control levels by Pepstatin A administration. Ca074-Me had no effect on CtsD activity assessed by fluorimetric activity in kidney lysates (Fig. 3A). The inhibitory effect of Ca074-Me on CtsB activity was demonstrated *in vivo* in 10 days UUO kidneys by IVIS analysis using a CtsB activable fluorescent probe (Fig. 3B). Morphometric analysis of Sirius Red, collagen III and IV cortical staining, showed an increase in fibrosis and thickening of the tubular basal membrane in injured kidneys (Fig. 3C–E). Ca074-Me administration had no effect over Sirius Red or collagen IV and only a moderate effect over collagen III. However, Pepstatin A treatment significantly reduced cortical accumulation of Sirius Red, collagen III and IV in fibrotic kidneys (Fig. 3C–E).

**Figure 4:**
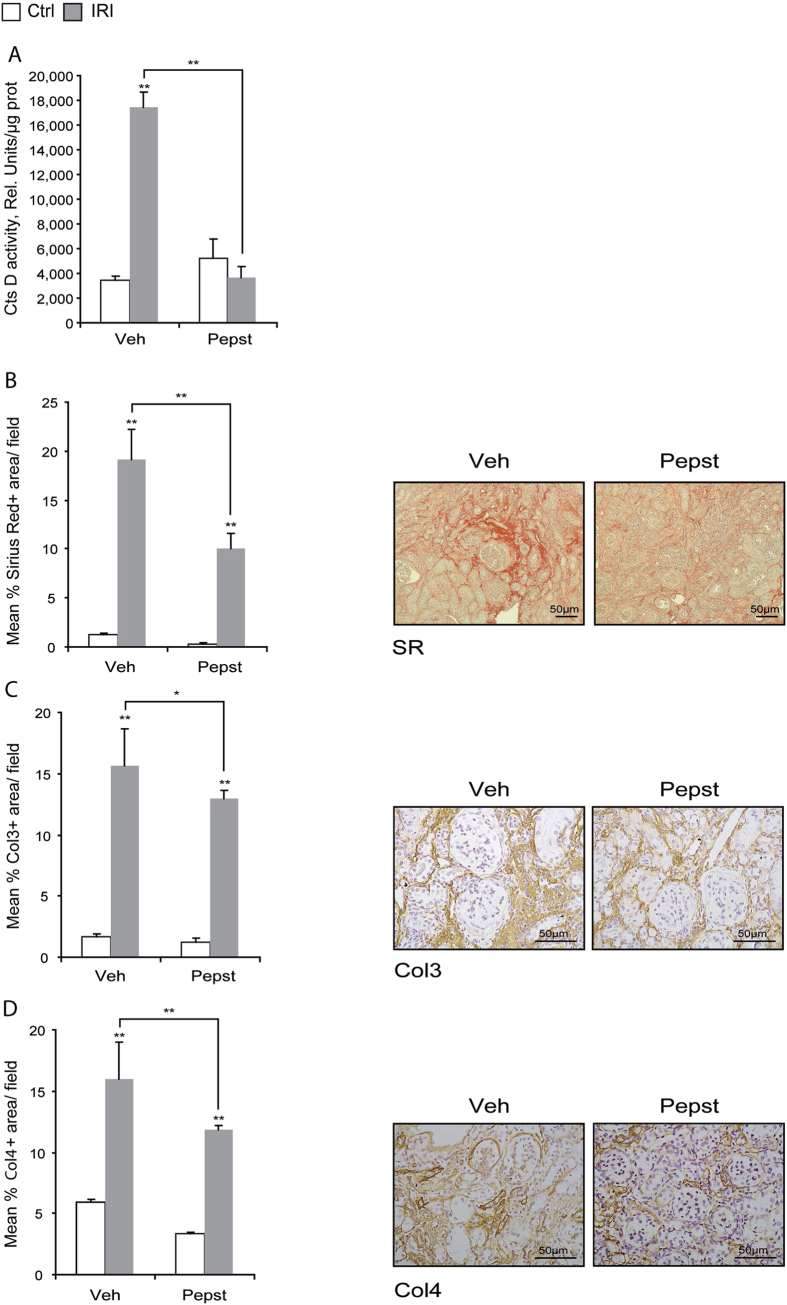
Cathepsin D inhibition reduces cortical collagen deposition in a chronic IRI model. CtsD fluorometric activity in kidney lysates assessed by the cleavage of a specific fluorescently labelled substrate **(A)**. Morphometric analysis and representative images of Sirius Red (SR) +ve **(B)**, Col3 +ve **(C)** and Col4 +ve **(D)** area/field in cortex of contralateral or IRI kidneys. Ischemia was performed for 35 minutes and kidneys were reperfused for 28 days. Animals were treated with vehicle or Pepstatin A 20 mg/Kg from day 5. N = 6, 1 way ANOVA, *P ≤ 0.05 or **P ≤ 0.01.

The effect of Pepstatin A on kidney fibrosis was confirmed in a second model of chronic ischemia reperfusion injury (IRI) (35 minutes of ischemia followed by 28 days of reperfusion). Pepstatin A was again administered from 5 days post-surgery. CtsD activity was significantly increased in IRI kidneys versus control and Pepstatin A treatment reduced this levels back to normal control as assessed by fluorimetric activity in kidney lysates (Fig. 4A). In agreement with the UUO results, Pepstatin A effectively reduced cortical accumulation of Sirius Red, collagen III and IV in IRI kidneys (Fig. 4B–D). Thus, CtsD but not B inhibition lead to a reduction in kidney fibrosis in two different models of chronic kidney disease.

### Pepstatin A reduces kidney fibrosis due to an increase in collagen degradation

**Figure 5:**
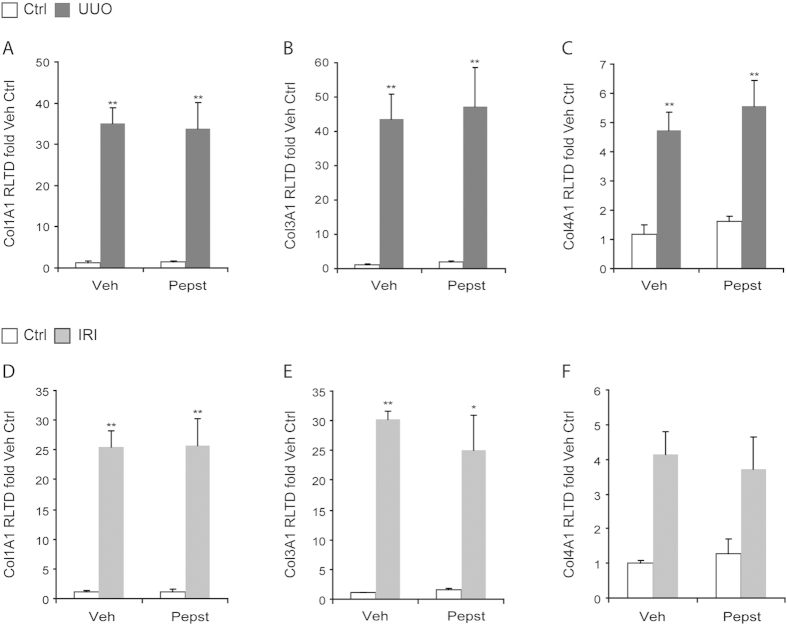
Pepstatin A does not affect kidney collagen transcription. Col1A1, Col3A1, Col4A1 mRNA expression of kidney cortex from contralateral and 15 days UUO kidneys **(A–C)** or contralateral and IRI kidneys **(D–F)**. UUO was performed for 15 days. Ischemia was performed for 35 minutes and kidneys were reperfused for 28 days. Animals were treated with vehicle or Pepstatin A 20 mg/Kg from day 5. N = 6, 1 way ANOVA, *P ≤ 0.05 or **P ≤ 0.01.

**Figure 6:**
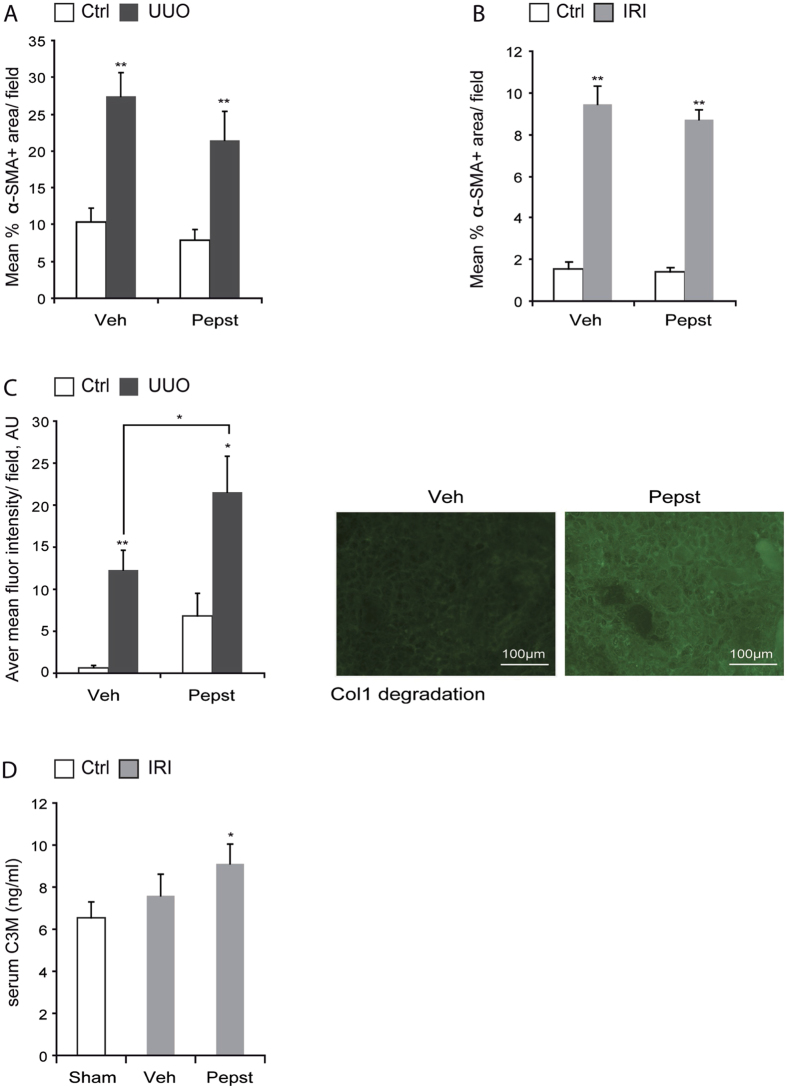
CtsD inhibition enhances collagen degradation with no changes in cortical myofibroblast numbers. Morphometric analysis of α-SMA +ve area/field of kidney cortex from contralateral and 15 days UUO kidneys **(A)** or contralateral and IRI kidneys **(B)**. Average mean fluorescent intensity and representative pictures of DQ collagen I degradation by *in situ* zymography of day 15 UUO kidneys treated with vehicle or Pepstatin A 20 mg/Kg **(C)**. Serum C3M ELISA in sham or IRI mice treated with vehicle or Pepstatin A 20 mg/Kg **(D)**. UUO was performed for 15 days. Ischemia was performed for 35 minutes and kidneys were reperfused for 28 days. Animals were treated with vehicle or Pepstatin A 20 mg/Kg from day 5. N = 6, for A and B, 1 way ANOVA, for (**C and D**), t-test, *P ≤ 0.05 or **P ≤ 0.01.

The reduction in collagen deposition with Pepstatin A treatment could be explained by changes in ECM gene transcription rate, myofibroblasts numbers or ECM degradation rate. Transcript levels of Col1A1, Col3A1 and Col4A1 were significantly increased in fibrotic kidneys in both models, however, the increase was not affected by Pepstatin A treatment (Fig. 5A–F). Pepstatin A treatment did not affect the cortical area occupied by myofibroblasts, determined by α-SMA immunostaining, in UUO or IRI kidneys (Fig. 6A,B). Finally, collagenolytic activity was analysed by *in situ* collagen I zymography in UUO cryosections and C3M peptide ELISA^13^ in sham and IRI serum. *In situ* zymography assesses cleavage of collagen I by using a specific DQ^TM^ collagen I fluorescein conjugated substrate. When this substrate is intact its signal is quenched by the close proximity of the dyes and only after enzyme-driven hydrolysis, there is an increase in fluorescence intensity. C3M is a collagen III proteolytic fragment, also called neo-peptide, derived from MMP mediated enzymatic cleavage. These peptides can be released from different tissues into circulation and are currently under study as possible biomarkers to assess collagen degradation in different diseases^14^. Indeed, C3M has already been reported as a marker for experimental renal fibrosis^15^. Both methods showed higher collagenolytic activities in Pepstatin A treated fibrotic mice (Fig. 6C,D). In summary, Pepstatin A effectively reduced fibrosis in two independent models of kidney fibrosis by enhancing collagenolytic activity as opposed to changes in the collagen synthesis or myofibroblast numbers.

### Pepstatin A activates the urokinase plasminogen activator system

**Figure 7:**
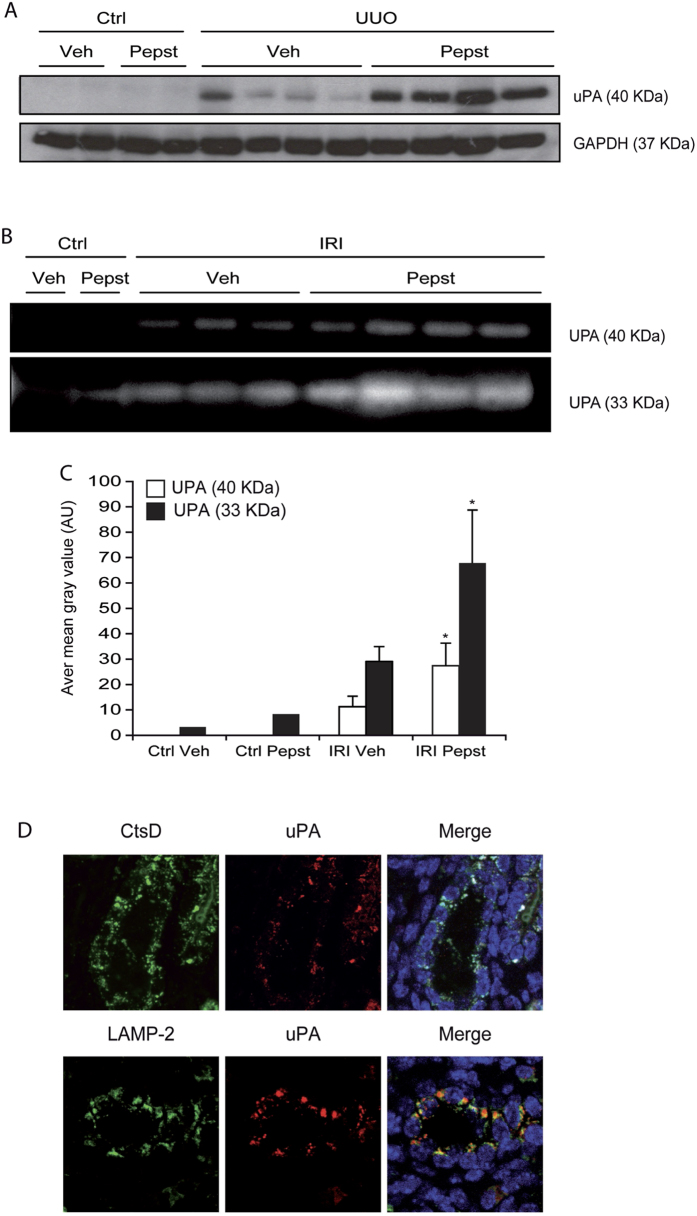
UPA expression is enhanced by Pepstatin A in renal fibrosis. UPA and GAPDH western blot **(A)** of contralateral or 15 days UUO kidney lysates. Plasminogen/casein zymography **(B)** with quantification below **(C)** in contralateral or IRI kidney lysates. Representative dual staining confocal pictures of CtsD/UPA or LAMP-2/UPA of a single epithelial tubular cell from a vehicle-treated UUO kidney **(D)**. CtsD or LAMP-2 (green), UPA (red) and merge with DAPI (blue). Confocal processing was performed by Image J software. UUO was performed for 15 days. Ischemia was performed for 35 minutes and kidneys were reperfused for 28 days. Animals were treated with vehicle or Pepstatin A 20 mg/Kg from day 5. N = 6, repeated measures of t-test, *P ≤ 0.05 or **P ≤ 0.01.

According to our results CtsD plays a pro-fibrogenic role in kidney fibrosis, as its inhibition enhances collagen degradation and reduces fibrosis. Cathepsins can enhance ECM turnover through activation of other proteases^16^. The urokinase plasminogen activator system plays a role in CKD by directly degrading ECM proteins and activating MMPs^17^. UPA protein expression was induced in fibrotic kidneys compared to the contralateral controls in UUO with a further increase after Pepstatin A administration (Fig. 7A). Plasminogen/casein zymography using kidney lysates from the IRI model confirmed the UUO results showing an increase in UPA activity in fibrotic kidneys from mice treated with Pepstatin A compared to vehicle (Fig. 7B,C).

Urokinase and its receptor (UPAR) are known to be in part regulated through endo/lysosomal recycling once UPA has bound its inhibitor PAI^17^. In vehicle treated fibrotic kidney (UUO and IRI) dual confocal immunofluorescence demonstrated co-localization of CtsD and UPA in a vesicle like pattern in the cytosol of tubular cells (Fig. 7D; Supplementary Fig. S2). Further confocal analysis of lysosomal-associated membrane protein-2 (LAMP-2), as a lysosomal marker, and UPA confirmed the vesicle like pattern as lysosomes (Fig. 7D; Supplementary Fig. S2). In summary, in our CKD models Pepstatin A is able to induce an increase in active urokinase. In addition to this, CtsD and UPA co-localize in lysosomes in fibrotic kidneys, pointing towards a possible interaction between UPA and CtsD within the lysosomes. Our results suggest that affectation of UPA endo/lysosomal recycling by CtsD inhibition, could lead to an increase of extracellular UPA, which could then modulate the activation of plasmin and potentially MMPs^18^,^19^ both of which enhance ECM degradation.

### CtsD inhibition enhances UPA secretion by impairing its endo/lysosomal recycling

**Figure 8:**
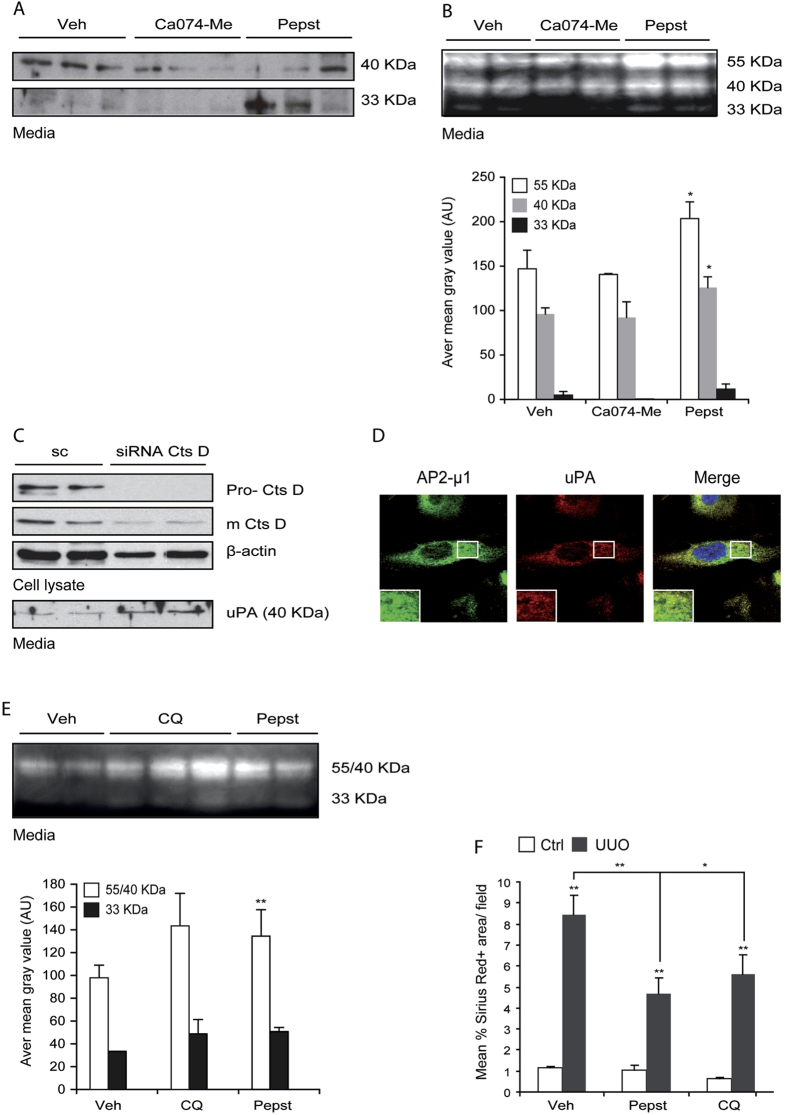
Pepstatin A enhances UPA secretion in human tubular epithelial cells by impairing its endo/lysosomal recycling. UPA and GAPDH WB **(A)** and plasminogen/casein zymography with quantification below **(B)** of concentrated media from HKC-8 cells treated with vehicle, 10μM Ca074-Me or 10μg/mL Pepstatin A for 48 hrs. CtsD and β-actin WB in cell lysate and UPA WB in media of hDTC transfected with scramble or CtsD siRNA for 48 hrs **(C)**. Representative dual staining confocal pictures of AP2-μ1/UPA in human distal tubular epithelial cells (hDTC) **(D)**. Plasminogen/casein zymography with quantification below **(E)** of concentrated media from HKC-8 cells treated with vehicle, 50 μM Chloroquine (CQ), 10μg/mL Pepstatin for 48 hrs. Experiments were repeated at least 3 times. Sirius Red (SR)+ve area/field **(F)** of kidney cortex of contralateral and 15 days UUO kidneys treated with vehicle, Pepstatin A 20mg/Kg or Chloroquine 20mg/Kg. N=6, repeated measures of t-test, *P ≤ 0.05 or **P ≤ 0.01.

Our results show co-localization of CtsD and UPA in lysosomes (Fig. 7D; Supplementary Fig. S2). UPA/UPAR complex is known to be recycled through endo/lysosomal internalization^17^. To investigate whether only CtsD or also CtsB has an effect on UPA lysosomal recycling, we treated the human tubular epithelial cell line HKC-8 with Pepstatin A and Ca074-Me. As expected only Pepstatin A but not Ca074-Me was able to inhibit CtsD activity (Supplementary Fig. S3A). UPA WB of concentrated media revealed an increase in extracellular UPA after Pepstatin A, but not Ca074-Me treatment (Fig. 8A). Plasminogen/casein zymography confirmed an enhancement of UPA activity by Pepstatin A showing three different isoforms of UPA, 55, 40 and 33 KDa^20^ (Fig. 8B). In agreement, CtsD siRNA transfection also showed an increased in UPA secretion into the extracellular media (Fig. 8C). Next we assessed whether Pepstatin A enhanced UPA secretion by affecting its endo/lysosomal recycling through clathrin-mediated endocytosis^21^. We analysed the UPA cellular distribution in primary human distal tubular epithelial cells (NCC^+^, cytokeratin-19^+^, collagen-1^−^, Supplementary Fig. S3B,C). We demonstrated co-localization of AP2-μ1 adaptor protein, which is essential for the clathrin vesicle formation, with UPA, suggesting that UPA was being endocytosed through the clathrin dependent pathway in hDTC (Fig. 8D). In order to impair endo/lysosomal activity we used the endo/lysosomal inhibitor, chloroquine (CQ). As with Pepstatin A treatment, HKC-8 cells treated with CQ showed increased levels of active UPA into the extracellular media by plasminogen/casein zymography (Fig. 8E). To investigate whether CQ had a similar effect to Pepstatin A *in vivo*, UUO was performed and animals were treated with Pepstatin A, CQ or vehicle. CQ endo/lysosomal inhibition is in part due to its ability to prevent endo/lysosomal acidification. This rise in pH will result in reduced lysosomal enzyme activity. CtsD activity in UUO kidney lysates from mice treated with CQ showed significantly less CtsD activity than vehicle treated UUO kidneys (Supplementary Fig. S3D). Consistent with our *in vitro* data, CQ was able to reduce cortical fibrosis to a similar level as Pepstatin A assessed by Sirius Red staining (Fig. 8F). Taken together our results suggest that Pepstatin A enhances UPA secretion by impairing its lysosomal recycling.

## Discussion

In this study we clearly demonstrate that inhibition of aspartyl cathepsin D leads to a reduction in interstitial fibrosis in two models of renal disease. The number of patients with CKD is increasing and some of these patients will progress onto end stage renal disease and face life-long dialysis or organ transplant. Treatment options for patients with progressive disease are limited, thus there is an urgent need to find new therapeutic targets that could lead to drug development. Interstitial fibrosis is almost invariably seen in patients with progressive CKD. Dysregulation of extracellular matrix (ECM) homeostasis leads to a gradual replacement of the healthy nephrons by electrondense fibrotic ECM. Proteases play a crucial role in regulating this process; however, our knowledge of proteases biology and function in CKD is still very limited.

Lysosomal proteases have been implicated in the pathogenesis of fibrotic disease in the liver, (CtsB and D)^6^,^7^ lung, (CtsK)^8^,^9^ and heart, (CtsL)^10^,^11^ but very little is known about their function in renal disease. The only reports relate to the role of cysteine but not aspartyl cathepsins (CtsD family group). There is decreased activity in the kidney of the cysteine cathepsins B, H and L accompanied by an increase in their urinary secretion in rat polycystic kidney disease^22^, puromycin induced nephrosis^23^ and rat and human diabetic nephropathy^24^,^25^. However, not all cysteine cathepsins decrease during proteinuric kidney disease and CtsL^26^ increases in proximal tubular cells and podocytes. Therefore, the role of cathepsins is currently far from understood, pointing towards a cell and disease specific function.

Screening of a panel of human renal biopsies show for the first time the expression of CtsD and B in distal and proximal tubular cells respectively in human renal disease (Fig. 1A,B). In agreement with Goto *et al.’s*^27^ observations in human normal kidney, CtsD is mainly expressed in distal convoluted tubules. Our results point towards an increase of CtsD expression in areas of tissue damage with no change in CtsB expression. The number of patients screened in our study was insufficient to draw statistical conclusions and further investigations will be needed in a bigger cohort to determine an association between level of CtsD expression and disease outcome.

To investigate the role of CtsD and B in CKD we used an aspartyl or cysteine protease inhibitor, Pepstatin A or Ca074-Me, in a murine CKD model, UUO. Pepstatin A but not Ca074-Me diminished collagen accumulation and thickening of the tubular basement membrane (Fig. 3C–E) with no effect on collagen transcription (Fig. 5A–C) or myofibroblast (Fig. 6A) numbers. Pepstatin A effects on fibrosis were reproduced in a second model of CKD, 35 minutes/28 days IRI (Figs 4B–D,5D,F and 6B). Our results suggest that Pepstatin A reduces fibrosis by increasing collagen I and III degradation (Fig. 6C,D).

Despite Pepstatin A being the best inhibitor against CtsD available and in contrast to Ca074-Me, which is a rather specific inhibitor against CtsB^28^, Pepstatin A can also affect other proteases of the same family, thus additional effects on other proteases cannot be excluded. Indeed, this may contribute to the outcome of our study, as redundancy and compensatory mechanisms^12^ are common problems when targeting only one member of a protease family. CtsD knock-out mice die approximately 26 days after birth due to neurological disorders^29^ replicating human deficiency^30^,^31^. In our models Pepstatin A did not completely block CtsD activity, achieving a reduction back to physiological levels (Figs 3A and 4A), avoiding possible undesirable secondary effects.

Cathepsins can indirectly modulate ECM turnover by affecting other proteases. We investigated the relationship between CtsD and UPA as an example of how CtsD can affect extracellular protease activity. Urokinase (UPA) was upregulated in fibrotic kidneys after the treatment with Pepstatin A (Fig. 7A–C). *In vitro*, extracellular UPA was increased in human tubular epithelial cells treated with CtsD but not B inhibitor and siRNA against CtsD (Fig. 8A–C).

UPA belongs to the urokinase plasminogen activator system, the role of which in CKD remains controversial^17^,^32^,^33^. UPA is anti-fibrotic in lung^34^ and liver^35^ whereas surprisingly no difference in the severity of UUO was observed in UPA knock-out mice^36^. UPA is secreted extracellularly and activated upon binding to its surface receptor, UPAR. Then activates other proteins, preferentially plasminogen into plasmin, which can directly degrade ECM proteins^37^,^38^,^39^ and also activates MMPs^18^,^19^, triggering further ECM degradation. Despite several reports in the literature of a direct link between UPA, plasmin and MMP activation, further work is required to demonstrate a link to CtsD.

PAI-1, UPA’s natural inhibitor, covalently binds to the UPA:UPAR complex inhibiting UPA’s enzymatic activity. The UPA:UPAR:PAI-1 complex is then rapidly internalized upon binding to LDL receptor-related protein-1 (LRP-1) through clathrin dependent endocytosis pathway into the lysosomes^21^. There UPA and PAI-1 are degraded and UPAR is recycled back to the cell surface. We confirmed localization of UPA in clathrin endosomal vesicles by co-localizing UPA with AP2-μ1 adaptor protein, which is an essential protein for the clathrin-coated pit formation, in hDTC (Fig. 8D). We also proved co-localization of CtsD and UPA within the lysosomes in fibrotic kidneys (Fig. 7D, Supplementary Fig. S2). Previous work by van Kasteren SI *et al.* support our hypothesis of Pepstatin A affecting endo/lysosomal recycling as they described EGFR clathrin dependent endo/lysosomal degradation being impaired by a cystatin-pepstatin inhibitor (CPI)^40^. In order to further confirm a link between lysosomal degradation and the increase in UPA, we used chloroquine (CQ) as endo/lysosome inhibitor. Both Pepstatin A and CQ had similar effects *in vitro*, increasing the active extracellular UPA (Fig. 8E). In addition, CQ administration in the UUO model mimicked the effect seen with Pepstatin A administration, reducing collagen accumulation and fibrosis (Fig. 8F).

In summary, here we report for the first time the distribution of CtsD and B in human renal disease and show the effect of their inhibition in two mouse models of renal fibrosis. We propose a novel mechanism by which CtsD inhibition by Pepstatin A leads to an increase in extracellular protease activity, in particular UPA, due to altered lysosomal recycling. This can trigger a proteolytic cascade activating first plasminogen into plasmin and culminating possibly with the regulation and activation of MMPs^18^,^19^. Both plasmin^37^,^38^,^39^ and MMPs are able to degrade ECM proteins causing a net reduction in fibrosis. This situation can be further sustained by a positive feedback loop, as plasmin is also able to activate UPA^20^. Our model does not exclude the regulation by CtsD of other proteases that might be recycled through the lysosomal pathway and further investigation will be needed to clarify this. This work opens new and exciting prospects for the treatment of CKD by targeting lysosomal proteases.

## Methods

### Reagents

CtsD fluorimetric activity assay was from Abcam. Pepstatin A, Chloroquine were from Sigma. Ca074-Me was from PeptaNova. Cat B 750 FAST Fluorescent Imaging Agent was from Perkin Elmer. DQ™ Collagen, type I from Bovine Skin, Fluorescein Conjugate was from Invitrogen Life Technologies. Human Plasminogen was from Biopur. Avidin/Biotin blocking kit, Citric acid based antigen unmasking solution, Vectastain Elite ABC Reagent, DAB peroxidase substrate kit, Vectashield mounting medium were purchased from Vector Laboratories. Unless otherwise reported all other reagents were from Sigma-Aldrich.

### Unilateral ureter obstruction (UUO) model of kidney fibrosis

All the animal studies were done in accordance to the UK Home Office regulations and under its approval (licence 60/4521). Left proximal ureter ligation was performed in 8–10 week C57BL/6 females. Right kidneys were used as controls. Animals were culled at 5, 7, 10 and 15 days post-surgery. 15 days UUO mice received intraperitoneal injections of vehicle, Pepstatin A (20 mg/Kg), Ca074-Me (10 mg/Kg) or Chloroquine (20 mg/Kg) from day 5 post-surgery three times a week up to 15 days. A minimum of 6 animals were used in each experimental group.

### Chronic ischemia reperfusion (IRI) model of kidney fibrosis

Ischemia was achieved by clamping the left renal pedicle for 35 minutes in 8–10 week C57BL/6 females. After 35 minutes the clamp was removed and the kidney reperfused for 28 days. Right kidneys were used as controls. Vehicle and Pepstatin A (20 mg/Kg) were administered by intraperitoneal injection from day 5 post-surgery three times a week up to 28 days. A minimum of 6 animals were used in each experimental group.

### Cathepsin B *in vivo* activity by IVIS imaging

For the IVIS analysis Cathepsin B Prosense 750 probe was injected intravenously according to manufacturer´s instructions in 10 days UUO mice. After 7 hours animals were anesthetized with isoflurane and scanned using 745 nm excitation and 800 nm emission wavelengths with an IVIS Spectrum CT (Caliper Life Sciences). Animals were scanned before injection and the background was subtracted. Data was analysed using Living Image 4.2 software, regions of interest (ROI) were drawn and the average of the Total Radiant Efficiency [p/s]/[μW/cm^2^] was calculated per ROI.

### Cell culture

HKC-8 cells^41^ or NRK-49F (ATCC^®^ CRL 1570™) were cultured in 1:1 Dulbecco’s modified Eagle’s: F12 medium or DMEM supplemented with 100 U/ml penicillin, 100 μg/ml streptomycin, 2 mM L-glutamine, 5% FBS, and maintained at 37 °C at an atmosphere of 5% CO_2_.

### siRNA transfection

During treatment cells were cultured in 0% FBS. Cells were transfected with 50 nM Scramble or 50 nM CtsD siRNA using INTERFERin (Polyplus) according to manufacturer’s instructions for 48 hrs. After that, media was collected and cell lysates were performed.

### Isolation of human primary distal tubular cells (hDTC)

Human kidneys cells were isolated from adult kidneys after surgical resection in accordance to the Research Ethics Committee guidelines and approval granted by the NRES Committee East Midlands-Derby (REC ref. 13/EM/0311), subject to patient consent. Briefly, after removing the kidney capsule, the cortex was mince and digested with 1 mg/ml collagenase IV for 60 minutes at 37 °C. Digested tissue was passed through a 40 μm cell strainer and centrifuged at 1200 rpm. Pellet was resuspended in RPMI, loaded in a two layer Percoll gradient (50%, 24.6%) and centrifuged at 3000 rpm for 25 minutes at 4 °C. Top layer containing DTC was washed twice in RPMI at 1200 rpm. DTC were seeded and maintained in DMEM/F-12, GlutaMAX™ supplemented with 100 U/ml penicillin, 100 μg/ml streptomycin, 2 mM L-glutamine, 10% FBS, and maintained at 37 °C at an atmosphere of 5% CO_2_. Cells were used in between passage 2 and 3. Phenotypic characterisation of the hDTC by WB confirmed they expressed thiazide-sensitive NaCl cotransporter (NCC)^+^ and cytokeratin-19^+^ but not Collagen 1^−^. Conversely, the rat kidney fibroblast cell line NRK-49F was positive for Collagen 1^+^ but did not express NCC^−^ or cytokeratin-19^−^ (Supplementary Fig. S3B). Microvilli were seen on scanning electron microscopy (Supplementary Fig. S3C). CtsD expression in hDTC was confirmed by WB (Supplementary Fig. S3B).

### Electron scanning microscopy

hDTC were cultured in 13 mm glass coverslips until full confluence, when they were fixed with 2% glutaraldehyde in Sorenson’s Phosphate Buffer overnight at 4 °C. Once fixed, they were washed and transferred into 100% ethanol and critically point dried. The cells were then gold coated and scanned in a VEGA3 TESCAN microscope.

### Immunohistochemistry in mouse and human samples

Immunohistochemistry was performed in 4 μm formalin-fixed kidney slides. Sections were deparaffinised and endogenous peroxidase was blocked with 2% hydrogen peroxide/methanol solution. Antigen retrieval was performed according to Supplementary Table S1. Endogenous avidin and biotin were blocked. Blocking solution and primary antibody was added overnight. After the addition of the secondary biotinilated antibody and ABC, sections were developed with DAB, counterstain with Mayer’s haematoxylin and mounted. For α-SMA, Collagen III and Collagen IV image analysis was performed at 200X using Nikon Eclipse Upright microscope and NIS-Elements BR Analysis software from Nikon. At least 10 random fields of cortex were analysed per kidney. For CtsD and B, human sections were assessed by an expert histopathologist (KMW). A minimum of three different patient biopsies were stained per disease.

### Study approval for human samples

Access to patient biopsies was approved by the NRES Committee East Midlands-Derby Research Ethics Committee (REC ref. 13/EM/0311). Normal, Minimal Change Disease, IgA nephropathy, FSGS, Diabetic nephropathy and ANCA associated vasculitis biopsy samples were taken under full ethical approval and patient consent, in accordance to the approved guidelines.

### Dual immunofluorescence

For sections, 4 μm formalin-fixed kidney tissue sections were deparaffinised. After microwave citrate saline antigen retrieval, permeabilization with 0.25% Triton X-100 was performed. Sections were then blocked with 5% BSA and primary antibodies, Thiazide-Sensitive NaCl Cotransporter (NCC), CtsD or LAMP-2 and UPA were applied in 1% BSA overnight. For coverslips, hDTC were cultured in glass coverslips, fixed with formalin and permeabilized with 0.1% saponine/0.5% BSA. After blocking with 3% BSA, primary antibodies AP2-μ1 and UPA were applied for 90 minutes. After washing, the secondary antibodies were sequentially added, donkey anti-rabbit Alexa 594 and anti-goat-FITC or goat anti-rat IgG Alexa 488. An extra blocking step with 20% rabbit serum was added between secondaries for the CtsD/UPA and the AP2-μ1/UPA dual stainings. Sections were finally mounted with ProLong^®^ Diamond DAPi conjugated mounting medium (Invitrogen) and analysed using a Leica TCS SP2 confocal microscope. Pictures were taken sequentially at 400X or 630X oil. 1.8 electronic zoom was made of a region of interest. 4 images of 1 μm slice each were acquired per section or 13 images of 0.5 μm slice each were acquired per coverslip. Image analysis was performed using Image J software. A detailed list of antibodies is provided in Supplementary Table S2.

### Plasminogen/casein zymography

Briefly, whole kidney lysates or concentrated medias mixed with 2× Laemmli buffer (Bio-Rad) were resolved in 10% acrylamide/bis-acrylamide gel containing 13 μg/mL of human plasminogen with EACA and 1 mg/mL of β-casein. Gel was washed twice with 2.5% Triton X-100 and incubated for 12–33 hrs at 37 °C in 0.1 M glycine soluction at pH: 8.0. The gel was stained with 0.2% Comassie R-250/ 10% acetic acid/45% methanol solution for 1 hr and distained with 7% acetic acid/ 40% methanol until optimal band resolution.

### *In situ* zymography

Kidney cryostat sections of 6 μm thickness where coated with low gelling temperature agarose containing 0.1 mg/mL DQ™ Collagen, type I fluorescein conjugated and 1 μg/mL of DAPI. Agarose was left to set in the fridge for 2 hrs. Then slides were incubated at 30 °C for 20 hrs. Fluorescence was detected using a Zeiss AxioImager microscope, 15 random pictures were taken at 200X. Fluorescent intensity was analysed using Image J software.

### CtsD activity assay

Cathepsin D activity was determined using a fluorescence-based assay “Cathepsin D activity assay” from Abcam. This assay utilizes the preferred cathepsin-D substrate sequence GKPILFFRLK(Dnp)-D-R-NH2) labelled with MCA. 0.1–10 μg of protein were used per assay according to manufacturer’s instructions. Assay plates were incubated at 37 °C and readings were performed in a fluorometer at 355 nm excitation and 520 emission for 2 hours. Results are expressed as a slope of fluorescence emission after 2 hours per μg of protein.

### Serum C3M Enzyme-linked immunosorbent assay

The specific MMP-generated neo-epitope of the collagen type III alpha 1 chain (C3M) was detected by competitive ELISA using a specific monoclonal antibody. Assay was performed as previously described^13^.

### Sirius Red staining

Kidneys were fixed with formalin, embedded in paraffin, and sections of 4 μm were routinely stained with 0.1% Sirius Red following standard procedures. Image analysis was performed as described in the immunohistochemistry section.

### SDS PAGE and immunoblotting

Total kidney or cell lysates were prepared with radioimmune precipitation assay buffer (RIPA). Concentrated medias were prepared using Amicon Ultra-0.5 Centrifugal Filters from Millipore. 10–30 μg of protein were fractionated in 8–12% SDS-PAGE and transferred into nitrocellulose membranes. Blots were incubated with 5% non-fatty dry milk or BSA in 0.1% TBS-Tween. Membranes were incubated overnight with primary antibodies, anti-Cathepsin D, anti-UPA, anti-α-SMA, and anti-GAPDH. Membranes were washed with TBS-Tween, incubated with secondary HRP conjugated antibodies and develop with ECL (Thermo scientific). A detailed list of antibodies is provided in Supplementary Table S2.

### RNA Isolation and Real Time PCR

RNA from kidney tissue was extracted with TriReagent. Briefly, RNA was precipitated from the aqueous phase with isopropanol and washed with 70% ethanol. Purity was assessed using a Nanodrop and cDNA synthesis was performed (Promega RT kit). Real time PCR was performed with SYBR Green JumpStart ready mix according to manufacturer’s instructions. Data was calculated using ∆∆Ct and 18S was used as a housekeeping gene. Data is plotted against vehicle control group. Primer sequences used are reported in the Supplementary Table S3.

### Statistics

Results are expressed as mean ± SEM unless otherwise stated in the figure legend. All p values were calculated using one way ANOVA followed by Bonferroni’s test or two tailed unpaired student’s t-test. *P ≤ 0.05 or **P ≤ 0.01 was considered statistically significant.

## Additional Information

**How to cite this article**: Fox, C. *et al.* Inhibition of lysosomal protease cathepsin D reduces renal fibrosis in murine chronic kidney disease. *Sci. Rep.* **6**, 20101; doi: 10.1038/srep20101 (2016).

## Supplementary Material

Supplementary Information

## References

[b1] Jha V. *et al.* Chronic kidney disease: global dimension and perspectives. Lancet 382, 260–272 (2013). 23727169 10.1016/S0140-6736(13)60687-X

[b2] National Kidney Foundation. KDOQI Clinical Practice Guideline for Diabetes and CKD: 2012 Update. Am J Kidney Dis 60, 850–886 (2012). 23067652 10.1053/j.ajkd.2012.07.005

[b3] Tan R. J. & Liu Y. Matrix metalloproteinases in kidney homeostasis and diseases. Am J Physiol Renal Physiol 302, F1351–1361 (2012). 22492945 10.1152/ajprenal.00037.2012 PMC3774496

[b4] Eddy A. A. Serine proteases, inhibitors and receptors in renal fibrosis. Thromb Haemost 101, 656–664 (2009). 19350108 PMC3136815

[b5] Moles A., Tarrats N., Fernandez-Checa J. C. & Mari M. Cathepsin B overexpression due to acid sphingomyelinase ablation promotes liver fibrosis in Niemann-Pick disease. J Biol Chem 287, 1178–1188 (2012). 22102288 10.1074/jbc.M111.272393 PMC3256848

[b6] Moles A., Tarrats N., Fernandez-Checa J. C. & Mari M. Cathepsins B and D drive hepatic stellate cell proliferation and promote their fibrogenic potential. Hepatology 49, 1297–1307 (2009). 19116891 10.1002/hep.22753 PMC2670444

[b7] Canbay A. *et al.* Cathepsin B inactivation attenuates hepatic injury and fibrosis during cholestasis. J Clin Invest 112, 152–159 (2003). 12865404 10.1172/JCI17740 PMC164289

[b8] Buhling F. *et al.* Pivotal role of cathepsin K in lung fibrosis. Am J Pathol 164, 2203–2216 (2004). 15161653 10.1016/S0002-9440(10)63777-7 PMC1615770

[b9] Srivastava M. *et al.* Overexpression of cathepsin K in mice decreases collagen deposition and lung resistance in response to bleomycin-induced pulmonary fibrosis. Respir Res 9, 54 (2008), 10.1186/1465-9921-9-54. 18638383 PMC2490691

[b10] Petermann I. *et al.* Lysosomal, cytoskeletal, and metabolic alterations in cardiomyopathy of cathepsin L knockout mice. FASEB J 20, 1266–1268 (2006). 16636100 10.1096/fj.05-5517fje

[b11] Tang Q. *et al.* Lysosomal cysteine peptidase cathepsin L protects against cardiac hypertrophy through blocking AKT/GSK3beta signaling. J Mol Med (Berl) 87, 249–260 (2009). 19096818 10.1007/s00109-008-0423-2

[b12] Guha S. & Padh H. Cathepsins: fundamental effectors of endolysosomal proteolysis. Indian journal of biochemistry & biophysics 45, 75–90 (2008). 21086720

[b13] Barascuk N. *et al.* A novel assay for extracellular matrix remodeling associated with liver fibrosis: An enzyme-linked immunosorbent assay (ELISA) for a MMP-9 proteolytically revealed neo-epitope of type III collagen. Clinical biochemistry 43, 899–904 (2010). 20380828 10.1016/j.clinbiochem.2010.03.012

[b14] Siebuhr A. S. *et al.* Matrix metalloproteinase-dependent turnover of cartilage, synovial membrane, and connective tissue is elevated in rats with collagen induced arthritis. Journal of translational medicine 10, 195 (2012), 10.1186/1479-5876-10-195. 22992383 PMC3551788

[b15] Papasotiriou M. *et al.* Serum and urine markers of collagen degradation reflect renal fibrosis in experimental kidney diseases. Nephrology, dialysis, transplantation 30(7), 1112–21 (2015). 10.1093/ndt/gfv063 25784725

[b16] Obermajer N., Jevnikar Z., Doljak B. & Kos J. Role of cysteine cathepsins in matrix degradation and cell signalling. Connective tissue research 49, 193–196 (2008). 18661341 10.1080/03008200802143158

[b17] Zhang G. & Eddy A. A. Urokinase and its receptors in chronic kidney disease. Front Biosci 13, 5462–5478 (2008). 18508599 10.2741/3093 PMC3142275

[b18] Han B., Nakamura M., Mori I., Nakamura Y. & Kakudo K. Urokinase-type plasminogen activator system and breast cancer (Review). Oncology reports 14, 105–112 (2005). 15944776

[b19] Deryugina E. I. & Quigley J. P. Cell surface remodeling by plasmin: a new function for an old enzyme. Journal of biomedicine & biotechnology 2012, 564259 (2012), 10.1155/2012/564259. 23097597 PMC3477900

[b20] Stepanova V. V. & Tkachuk V. A. Urokinase as a multidomain protein and polyfunctional cell regulator. Biochemistry (Mosc) 67, 109–118 (2002). 11841346 10.1023/a:1013912500373

[b21] Czekay R. P., Kuemmel T. A., Orlando R. A. & Farquhar M. G. Direct binding of occupied urokinase receptor (uPAR) to LDL receptor-related protein is required for endocytosis of uPAR and regulation of cell surface urokinase activity. Mol Biol Cell 12, 1467–1479 (2001). 11359936 10.1091/mbc.12.5.1467 PMC34598

[b22] Schaefer L., Han X., Gretz N. & Schaefer R. M. Alterations of cathepsins B, H and L in proximal tubules from polycystic kidneys of the Han:SPRD rat. Kidney Int 50, 424–431 (1996). 8840269 10.1038/ki.1996.332

[b23] Huang S. *et al.* Suppressed activities of cathepsins and metalloproteinases in the chronic model of puromycin aminonucleoside nephrosis. Kidney Blood Press Res 22, 121–127 (1999). 10394110 10.1159/000025917

[b24] Peres G. B., Juliano M. A., Simoes M. J. & Michelacci Y. M. Lysosomal enzymes are decreased in the kidney of diabetic rats. Biochim Biophys Acta 1832, 85–95 (2013). 23032151 10.1016/j.bbadis.2012.09.011

[b25] Shechter P., Boner G. & Rabkin R. Tubular cell protein degradation in early diabetic renal hypertrophy. J Am Soc Nephrol 4, 1582–1587 (1994). 8025232 10.1681/ASN.V481582

[b26] Sever S. *et al.* Proteolytic processing of dynamin by cytoplasmic cathepsin L is a mechanism for proteinuric kidney disease. J Clin Invest 117, 2095–2104 (2007). 17671649 10.1172/JCI32022 PMC1934589

[b27] Goto M., Mizunashi K., Kimura N. & Furukawa Y. Decreased sensitivity of distal nephron and collecting duct to parathyroid hormone in pseudohypoparathyroidism type I. J Am Soc Nephrol 12, 1965–1970 (2001). 11518791 10.1681/ASN.V1291965

[b28] Murata M. *et al.* Novel epoxysuccinyl peptides. Selective inhibitors of cathepsin B, *in vitro*. FEBS letters 280, 307–310 (1991). 2013328 10.1016/0014-5793(91)80318-w

[b29] Koike M. *et al.* Cathepsin D deficiency induces lysosomal storage with ceroid lipofuscin in mouse CNS neurons. J Neurosci 20, 6898–6906 (2000). 10995834 10.1523/JNEUROSCI.20-18-06898.2000 PMC6772823

[b30] Steinfeld R. *et al.* Cathepsin D deficiency is associated with a human neurodegenerative disorder. Am J Hum Genet 78, 988–998 (2006). 16685649 10.1086/504159 PMC1474096

[b31] Siintola E. *et al.* Cathepsin D deficiency underlies congenital human neuronal ceroid-lipofuscinosis. Brain 129, 1438–1445 (2006). 16670177 10.1093/brain/awl107

[b32] Zheng G. & Harris D. C. Plasmin in renal interstitial fibrosis: innocent or guilty? Kidney Int 66, 455–456 (2004). 15200456 10.1111/j.1523-1755.2004.00811.x

[b33] Eddy A. A. & Fogo A. B. Plasminogen activator inhibitor-1 in chronic kidney disease: evidence and mechanisms of action. J Am Soc Nephrol 17, 2999–3012 (2006). 17035608 10.1681/ASN.2006050503

[b34] Hattori N. *et al.* The plasminogen activation system reduces fibrosis in the lung by a hepatocyte growth factor-dependent mechanism. Am J Pathol 164, 1091–1098 (2004). 14982862 10.1016/S0002-9440(10)63196-3 PMC1614722

[b35] Lieber A. *et al.* Adenovirus-mediated urokinase gene transfer induces liver regeneration and allows for efficient retrovirus transduction of hepatocytes *in vivo*. Proc Natl Acad Sci USA 92, 6210–6214 (1995). 7597103 10.1073/pnas.92.13.6210 PMC41672

[b36] Yamaguchi I. *et al.* Endogenous urokinase lacks antifibrotic activity during progressive renal injury. Am J Physiol Renal Physiol 293, F12–19 (2007). 17356128 10.1152/ajprenal.00380.2006

[b37] Mackay A. R., Corbitt R. H., Hartzler J. L. & Thorgeirsson U. P. Basement membrane type IV collagen degradation: evidence for the involvement of a proteolytic cascade independent of metalloproteinases. Cancer research 50, 5997–6001 (1990). 2144209

[b38] Law R. H., Abu-Ssaydeh D. & Whisstock J. C. New insights into the structure and function of the plasminogen/plasmin system. Current opinion in structural biology 23, 836–841 (2013). 24252474 10.1016/j.sbi.2013.10.006

[b39] Huang Y., Border W. A., Lawrence D. A. & Noble N. A. Noninhibitory PAI-1 enhances plasmin-mediated matrix degradation both *in vitro* and in experimental nephritis. Kidney Int 70, 515–522 (2006). 16788698 10.1038/sj.ki.5000353

[b40] van Kasteren S. I. *et al.* A multifunctional protease inhibitor to regulate endolysosomal function. ACS chemical biology 6, 1198–1204 (2011). 21910425 10.1021/cb200292c PMC3220280

[b41] Racusen L. C. *et al.* Cell lines with extended *in vitro* growth potential from human renal proximal tubule: characterization, response to inducers, and comparison with established cell lines. The Journal of laboratory and clinical medicine 129, 318–329 (1997). 9042817 10.1016/s0022-2143(97)90180-3

