# Supplementary material for: Inhibition of lysosomal protease cathepsin D reduces renal fibrosis in murine chronic kidney disease
Source: Sci Rep. 2016 Feb 2;6:20101. doi: 10.1038/srep20101 (PMC4735715; doi:10.1038/srep20101)
Supplement: Supplementary Information [file srep20101-s1.pdf]

Inhibition of lysosomal protease cathepsin D reduces renal fibrosis in murine chronic kidney disease

Christopher Fox, Pasquale Cocchiaro, Fiona Oakley, Rachel Howarth, Krystena Callaghan, Jack Leslie, Saimir Luli, Katrina M Wood, Federica Genovese, Neil S Sheerin, Anna Moles*

Supplementary Figure S1: Cathepsin D expression in UUO time-course

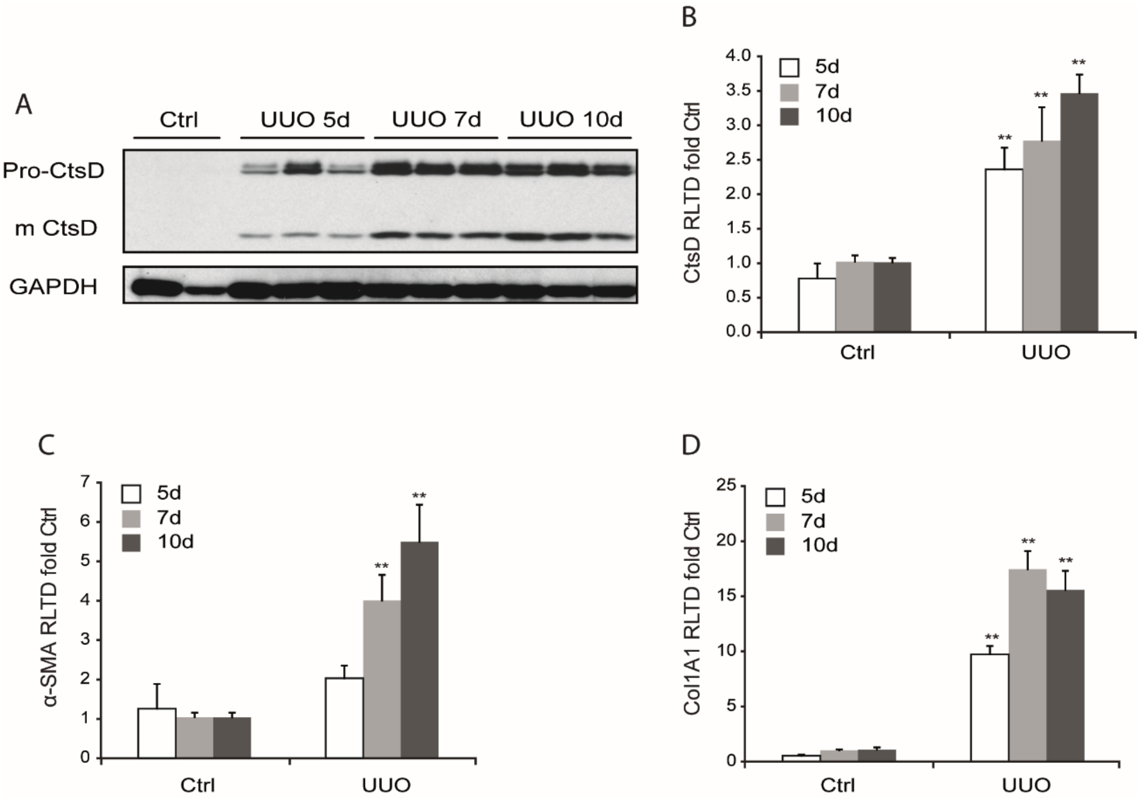

Supplementary Figure S2: Cathepsin D and UPA co-localize within the lysosomes in fibrotic IRI kidneys

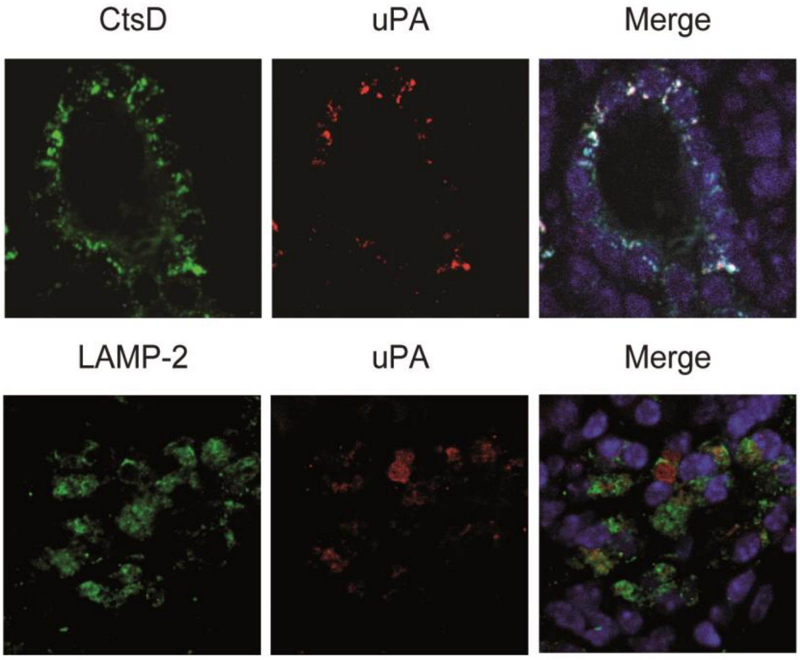

Supplementary Figure S3: Pepstatin A enhances UPA secretion by human tubular epithelial

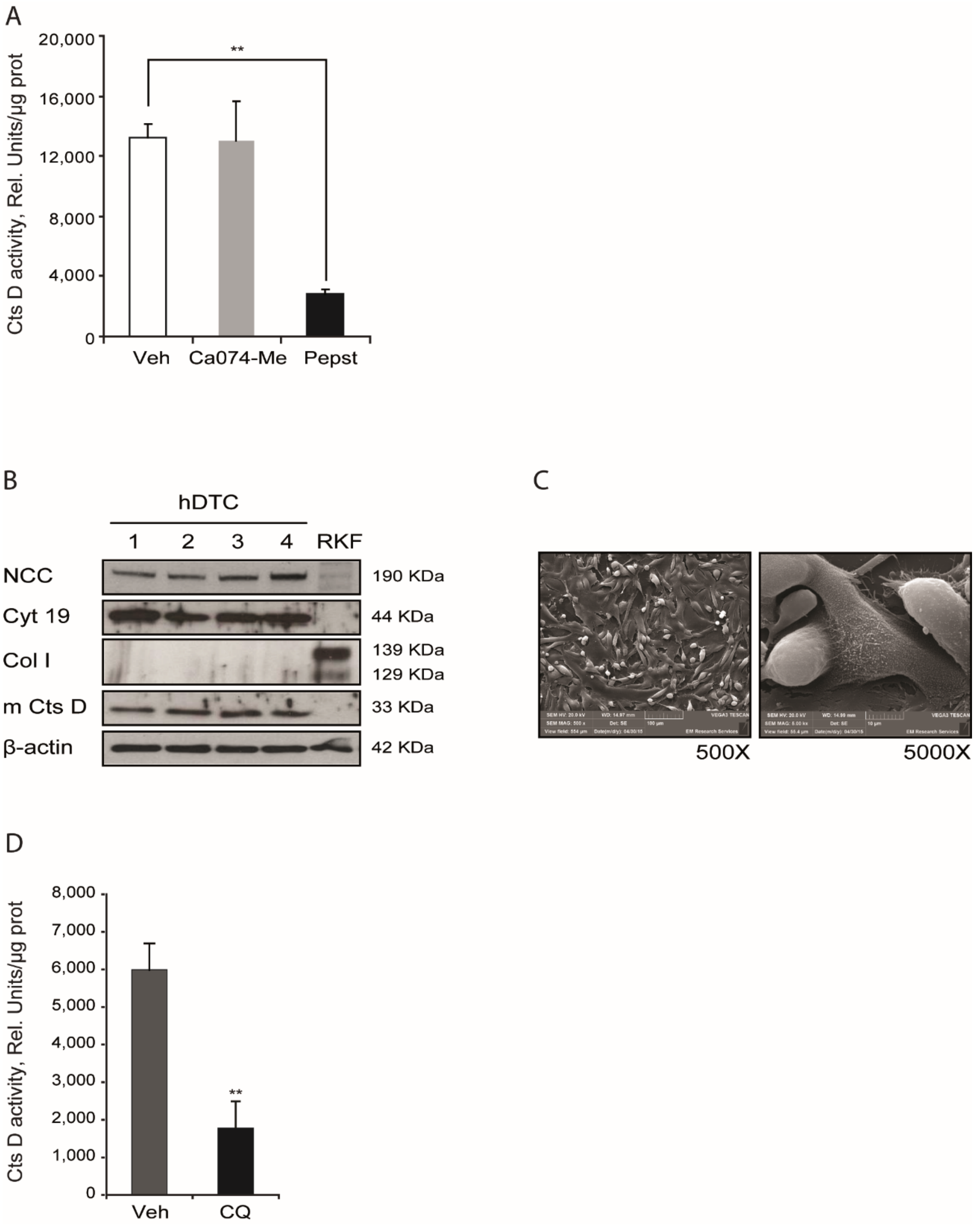

SUPPLEMENTARY FIGURE LEGENDS

Supplementary Figure S1: Cathepsin D expression in UUO time course

CtsD and GAPDH western blot **(A)** from kidney lysates. CtsD, α-SMA, Col1A1 mRNA expression **(B, C, D)** from contralateral and 5, 7, 10 days UUO kidneys. N=6, t-test, $^{*}P \le 0.05$ or $^{**}P \le 0.01$.

Supplementary Figure S2: Cathepsin D and UPA co-localize within the lysosomes in fibrotic IRI kidneys

Representative dual staining confocal pictures of CtsD/UPA or LAMP-2/UPA of a single epithelial tubular cell from fibrotic vehicle IRI kidneys sections. CtsD or LAMP-2 (green), UPA (red) and merge with DAPI (blue). Confocal processing was performed by Image J software. In this study ischemia was performed for 35 minutes and kidneys were reperfused for 28 days. Animals were treated with vehicle or Pepstatin A 20mg/Kg from day 5. N=6, 1 way ANOVA, $^{*}P \le 0.05$ or $^{**}P \le 0.01$.

Supplementary Figure S3: Pepstatin A enhances UPA secretion by human tubular epithelial

CtsD fluorometric activity assay in HKC-8 cell lysates **(A)** from HKC-8 cells treated with vehicle, 10μM Ca074-Me or 10μg/mL Pepstatin A for 48 hrs. Anti-Thiazide-Sensitive NaCl Cotransporter (NCC), Cytokeratin-19, Collagen type 1, CtsD and β-actin Western Blot in hDTC lysates from 4 different preparations at passage 2 and NRK-49F rat fibroblast cell line (RKF) lysates **(B)**. Representative electron microscopy images of hDTC at passage 3 **(C)**. Pictures were taken at 500X and 5000X. CtsD fluorometric activity in kidney lysates of 15 days UUO

vehicle and chloroquine treated mice assessed by the cleavage of a specific fluorescently labelled substrate **(D)**. Experiments were repeated at least 3 times. t-test, $*P \leq 0.05$ or $**P \leq 0.01$.

SUPPLEMENTARY TABLES

Supplementary table 1: Detailed instructions for immunohistochemistry staining

Antigen retrieval	Primary antibody
20µg/mL Proteinase K	Rabbit polyclonal Collagen IV (ab19808, Abcam)
20µg/mL Proteinase K	Goat anti-collagen III (1330-01, Southern Biotech)
Sodium citrate antigen unmasking solution	Monoclonal Anti-Actin, α-Smooth Muscle (F3777, Sigma- Aldrich)
Sodium citrate antigen unmasking solution	Rabbit polyclonal anti-Cathepsin D (sc10725, St Cruz Biotechnology)
Sodium citrate antigen unmasking solution	Rabbit polyclonal anti-Cathepsin B (NBP1-19797, Novus Biologicals)

Supplementary table 2: Primary antibody list

Dual immunofluorescence	WB
AP-2µ1 (sc-49150, St Cruz)	α-SMA (A5228, Sigma)
Aquaporin-1 (sc-9878, St Cruz)	β-actin (A5316, Sigma)
CtsB (sc-13985, St Cruz)	CtsB (sc-13985, St Cruz)
CtsD (sc-6486, St Cruz)	CtsD (sc-6486, St Cruz)
LAMP-2 (ab13524, Abcam)	CtsL (ab133641, Abcam)
Anti-Thiazide-Sensitive NaCl Cotransporter or NCC (AB3553, Merck Millipore)	GAPDH (ab22555, Abcam)
UPA (sc-14019, St Cruz)	UPA (sc-14019, St Cruz)

Supplementary table 3: Mouse primer sequences

Gene (GenBank Accession)	Primer sequence
α-SMA (NM_007392)	Fw: 5'-TCAGCGCCTCCAGTTCCT-3'
	Rv: 5'-AAAAAAAACCACGTAACAAATCAA-3'
Col1A1 (NM_007742)	Fw: 5'-TTCACCTACAGCACGCTTGTG-3'
	Rv: 5'-GATGACTGTCTTGCCCCAAGTT-3'
Col 3A1 (NM_009930)	Fw: 5'-CTGTAACATGGAAACTGGGGAAA-3'
	Rv: 5'-CCATAGCTGAACTGAAAACCACC-3'
Col 4A1 (NM_009931)	Fw: 5'-TCCGGGAGAGATTGGTTTCC-3'
	Rv: 5'-CTGGCCTATAAGCCCTGGT-3'
CtsB (NM_007798)	Fw: 5'-TCCTTGATCCTTCTTTCTTGCC-3'
	Rv: 5'-ACAGTGCCACACAGCTTCTTC-3'
CtsD (NM_009983)	Fw: 5'-GCTTCCGGTCTTTGACAACCT-3'
	Rv: 5'-CACCAAGCATTAGTTCTCCTCC-3'
CtsL (NM_009984)	Fw: 5'-TCGGTGACATGACCAATGAGG-3'
	Rv: 5'-AGCGGTTCCTGAAAAAGCCT-3'
Cystatin B (NM_007793)	Fw: 5'-AGGTGAAGTCCCAGCTTGAAT-3'
	Rv: 5'- GTCTGATAGGAAGACAGGGTCA-3'
Cystatin C (NM_009976)	Fw: 5'-AGGAGGCAGATGCCAATGAG-3'
	Rv: 5'-GGGCTGGTCATGGAAAGGA-3'
